# Functional Outcome of Midclavicular Fracture Fixation Utilising a Reconstruction Plate

**DOI:** 10.5704/MOJ.1311.008

**Published:** 2013-11

**Authors:** Elidrissi Mohammed, H Mahadane, A Mechchat, M Shimi, A Elibrahimi, A Elmrini

**Affiliations:** Department of Orthopeadic Surgery B4, Hassan II University Hospital, Fez, Morocco; Department of Orthopeadic Surgery B4, Hassan II University Hospital, Fez, Morocco; Department of Orthopeadic Surgery B4, Hassan II University Hospital, Fez, Morocco; Department of Orthopeadic Surgery B4, Hassan II University Hospital, Fez, Morocco; Department of Orthopeadic Surgery B4, Hassan II University Hospital, Fez, Morocco; Department of Orthopeadic Surgery B4, Hassan II University Hospital, Fez, Morocco

## Abstract

Background: Clavicular fractures are common injuries. The aim of this study is to present results of surgical treatment of midclavicular fracture managed with open reduction and internal fixation (ORIF) with superior reconstruction plating. This study involved 34 patients, with mean age of 31 years, with completely displaced midclavicular fractures, 28 on the right and 6 on the left, stabilized by contoured plate. One patient developed an early wound infection, which was successfully managed by surgical debridement. The average time of union was 14 weeks, with one case of nonunion. The average constant score was 95.33 with SD 3.4 in one year follow up. Plate fixation of completely displaced midshaft clavicular fracture provides stable fixation and improves the functional outcome.

**Key Words:**

Clavicle fracture, Open reduction and internal fixation, conservative treatment, surgical treatment

## Introduction

The clavicle fracture accounts for 2.6–4% of all fractures and between 35% and 44% of all injuries to the shoulder girdle^1^-^4^. Seventy to eighty percent of these fractures occur in the midshaft^5^. Traditionally the fracture has been treated nonoperatively, even when substantial displacement has been present^6^,^7^. The consensus of management is inclining towards open reduction and internal fixation for displaced midclavicular fractures, as the conservative management gives poor results^8^. Clavicular plating remains the gold standard of operative treatment^9^. Other types of internal fixation that have been used include intramedullary devices (titanium elastic nails), Rockwood pins, Kirschner wire, Rush nail and Kuntscher nails. However, most of these implants went into disrepute because of implant-related problems requiring removal of implants after fracture union^10^,^11^.

The aim of this study is to present the outcome of surgical treatment of midshaft clavicular fracture in adults by open reduction and internal fixation with superior reconstruction plating.

The objective of this study was to determine survivorship, functional outcome and failure modes among patients with bone tumours of the distal femur who underwent tumor excision and reconstruction using tumor endoprosthesis at the UP-MuST Unit of the Philippine General Hospital.

## Patients and Methods

This retrospective study involved thirty four patients with completely displaced midclavicular fractures who underwent open reduction and internal fixation with superior plating in Fez university hospital between January 2009 and June 2012. The study includes all patients with completely displaced midclavicular fractures, whatever the type of fracture (transverse, oblique, or comminuted). Demographic variables, mode of injury, injury-surgery interval, hospital stay, and time required for union were recorded. The fracture was classified according to Allman system. The displacement was evaluated using an anteroposterior X-ray view of the shoulder.

All operations were performed under general anaesthesia; the patient was placed supine in the beach-chair position, with the head turned and neck flexed away from the side of the operation. The incision was made over the anterosuperior aspect of the clavicle. The fracture was reduced and stabilized by a contoured 3.5 mm plate placed superiorly with at least 6 cortical purchases on either side of the fracture. Interfragmentary screws were inserted as deemed necessary. The limb was supported with an arm sling post operatively, and check x-ray done at two weeks. The patient was advised to carry out gentle pendulum exercises only. Union was considered to have occurred if clinically the fracture site was non-tender and, no abnormal movement was demonstrable, and radiologically when callus was visible.

## Results

This study includes thirty four patients, 32 male and 2 female, with the mean age of 31 years (+/-9) [20, 58]. Twenty-eight clavicles were fractured on the right side and 6 on the left. The mechanism was direct force in 10 cases, and indirect in all the others. All patients ware admitted to the emergency department immediately after the injury. There were three patients with associated lesions, one with an ipsilateral fractures of the humerus and both forearm bones the second had an associated upper humeral extremity fracture, and the third had a fracture of the femur. The mean operation deadline was 48 hours. One patient developed an early wound infection, which was successfully managed by surgical debridement.

All patients were followed up until clinical and radiological union. Radiological union was defined as visible bridging callus or absence of a visible fracture line. The average time of union was 14 weeks (12-20 weeks). There was one case of nonunion; a revision procedure was performed, using a DCP plate with iliac crest autograft and the fracture united 17 weeks later. The average Constant Shoulder Score was 95.33 with SD 3.4 in one year follow up. All the patients were relatively satisfied with the procedure. None of the patients had implant loosening or implant failure. Removal of implant was carried out in seven patients, for protrusion in four patients and following patient requests in three patients.

## Discussion

Fractures of the clavicle are common, accounting for 2.6% of all fractures. More than 75% of them are located in the midshaft 12. Most have a good outcome few or no residual symptoms once the fracture had healed 13,14 and the overall incidence of nonunion is less than 1%^15^,^16^. The management of displaced clavicle fractures has undergone recent transition 17. It was traditionally treated nonoperatively, with the expectation that little functional loss will result, despite substantial residual radiographic malalignment^18^,^19^,^20^, ^21^. Many conservative treatment methods have been described, but simple arm sling or figures of 8 bandage have been widely used 22. Neither technique reduces the fracture, the outcomes were identical, but arm sling demonstrated better patient satisfaction 23. However, more recent studies of displaced midshaft clavicular fractures have shown a nonunion rate of 15% in one series as well as a rate of unsatisfactory patientoriented outcomes of 31% in one report and 32%, in another, which are much higher rates than previously reported^24^,^25^,^26^, ^27^.

Neer reported nonunion in only three of 2235 patients with midclavicular fractures treated by closed methods, while Rowe reported nonunion in four of 566 clavicular fractures. This information dominated the clinical approach to displaced clavicular fractures. These studies also suggested a higher nonunion rate with operative care 27. These previous studies depended on surgeon or radiograph-based outcome measures that may not have detected subtle deficits. Previously, malunion of the clavicle was thought to be of radiographic interest only and required no treatment. However, it is becoming increasingly apparent that clavicular malunion is a distinct clinical entity with radiographic, orthopaedic, neurologic, and cosmetic features. Nowak et al. examined the late sequelae in 208 adult patients with clavicular fractures and found that, at ten years after the injury, ninety-six patients (46%) still had symptoms despite the fact that only fifteen (7%) had nonunion 28. There is increasing evidence that patients can have substantial dissatisfaction following a clavicular malunion because of symptoms including weakness and easy fatigability, especially with overhead work. Mckee et al have demonstrated that abduction endurance was the most negatively affected muscle strength. This finding may explain the trend toward a higher prevalence of patient dissatisfaction with increasing clavicular shortening. They concluded also that there was some variability in the features of clavicular malunion, shortening in the medial-lateral dimension with inferior displacement and anterior rotation of the lateral fragment seen in most cases. It is reasonable to conclude that shortening in the coronal plane has a negative effect on muscle-tendon tension and muscular balance 29. The reported results in previous studies regarding shoulder functions after shortened but united midshaft clavicle fractures are controversial. Lazarides and Zafiropoulos reported that shortening of more than 18 mm in male patients and 14 mm in female patients was associated with poor clinical outcome 30. Ledger et al.,^31^ Eskola et al., 32 Hill et al.^33^ and Wick et al. 34 also reported - poor clinical outcome if the shortening was more than 15 or 20 mm. A recent randomized clinical trial by the Canadian Orthopedic Trauma Society showed that early primary plate fixation of completely displaced midshaft clavicular fractures resulted in improved patient-oriented outcomes, improved surgeonoriented outcomes, earlier return to function, and decreased rates of nonunion and malunion. There were no catastrophic complications in the operative group such as brachial plexus palsy, vascular injury, or pneumothorax; implant removal was the most common reason for reintervention. Patients were more satisfied with the shoulder (and its appearance) following operative intervention 27. We found a few studies that insisted that conservative treatment can be carried out in midshaft clavicle fractures with a shortening of 20 mm or more. Rasmussen et al. 35 Ristevski et al. have demonstrated that patients with a degree of malunion following a clavicle fracture may have scapular malalignment. These patients have clinically evident shoulder ptosis, a ‘‘driven in’’ or medially translated shoulder, and a prominent inferomedial border of the scapula. The acromion closely follows the distal clavicular fragment and translates medially, inferiorly, and anteriorly. The translations of the superior and inferior angles of the scapula are quite variable in magnitude and direction, and on average, these angles translate substantially less than in the acromion. Correlation can exist between the degree of scapular malalignment and shoulder dysfunction^36^.

In our series, there was one case of nonunion, excellent function with average Constant score of 95.33 and all fractures had united in 14 weeks or less. Although the complication rate of 34% and a re-operation rate of 18% (most for implant removal) are reported in the operative group, in our series we encountered complications in one case, and seven reoperations; all for implant removal. The complications related to plate fixation are infection, plate failure, hypertrophic or dysesthetic scars, implant loosening, non union, and rarely; intraoperative vascular injury 37,38. No early complications occurred after implant removal. We believe that clavicular plating of displaced midclavicular fractures is a good and efficient treatment.

**Fig. 1:**
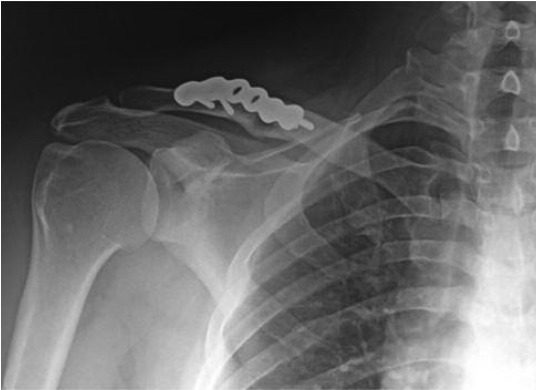
: Radiological control at 10 months - follow up of a right midclavicular fracture treated by reconstruction plate.

## Conclusion

Early primary plate fixation of completely displaced midshaft clavicular fractures has an improved outcome. We advocate superior placement of the plate with six cortical purchases on either side of the fracture for a stable construct with predictable union.
